# Healed Perforated Corneal Ulcers in Human

**DOI:** 10.3390/life15060939

**Published:** 2025-06-11

**Authors:** Yasser Helmy Mohamed, Masafumi Uematsu, Mao Kusano, Keiji Suzuki, Akio Oishi

**Affiliations:** 1 Department of Ophthalmology and Visual Sciences, Graduate School of Biomedical Sciences, Nagasaki University, Nagasaki 852-8501, Japan; yasserhelmy@nagasaki-u.ac.jp (Y.H.M.); maok@ngasaki-u.ac.jp (M.K.); akio.oishi@nagasaki-u.ac.jp (A.O.); 2 Department of Radiation Medical Sciences, Atomic Bomb Disease Institute, Nagasaki University, Nagasaki 852-8131, Japan; kzsuzuki@nagasaki-u.ac.jp

**Keywords:** perforated corneal ulcer, healing, myofibroblasts, keratocytes, migration, remodeling

## Abstract

This study investigates the pathophysiological process of healed perforated corneal ulcers (HPCUs) in humans. All subjects underwent keratoplasty due to opacities or leakage from HPCUs. Half of each specimen was fixed with 4% glutaraldehyde for transmission electron microscope (TEM) examination. The other half was fixed in 10% formaldehyde for immunofluorescence (IF) examination. TEM identified layered structures with two cell types (polygonal and elongated) connected by gap or adherent junctions during early stage of healing. Both apoptotic and mitotic changes were found in both types of cells. There were no endothelial cells or Descemet’s membrane (DM) present in early stage of healing. During the intermediate stage, the healed area comprised three layers: epithelium, Bowman’s layer, and stroma, with an increase in stromal collagen. Later, adjacent endothelial cells crept in, forming DM and completing the cornea’s 5-layer structure. IF examinations revealed that vimentin^+^ and α-smooth muscle actin (αSMA)^+^ myofibroblasts gathered around the damaged site. Proliferating cell nuclear antigen^+^ cells, which indicated cell proliferation, were found in both cells. Anti-phospho-histone H2AX antibodies were found in some epithelial cells. CK14-positive cells were only found in superficial polygonal cells. Corneal wound healing is a complex process that includes apoptosis, cell migration, mitosis, differentiation, and extracellular matrix remodeling.

## 1. Introduction

The cornea, which constitutes approximately 70% of the eye’s refractive power, ensures that light is focused and transmitted without scattering through the lens and onto the retina. Maintaining the tissue integrity of the cornea is crucial for clear vision, as eye injuries can cause permanent damage, leading to opacification and reduced visual acuity [1]. While superficial corneal scars can be treated relatively well, deep corneal scars or endothelial diseases often require corneal transplantation as the primary option in order to restore clear vision. Unfortunately, due to the scarcity of corneal donor tissue and limited access to corneal surgery, corneal opacification remains a significant cause of blindness worldwide [2,3].

The corneal wound healing process, which can result in stromal opacity, occurs following injury, surgery, or infection of the cornea. After epithelial and stromal damage, a complex sequence of events contributes to repairing the cornea and restoring its normal structure and function [4].

Therefore, understanding corneal wound healing is not only relevant to basic science, but it is also crucial for effective medical management. Frequent traumatic injuries to the cornea and the rising number of refractive surgeries highlight the importance of advancing our knowledge of corneal healing mechanisms and finding efficient ways to accelerate and enhance wound healing [5].

Following injury, corneal stromal keratocytes transform into an activated repair phenotype of stromal fibroblasts, which then actively participate in the wound healing process [6,7]. Fibroblasts can additionally undergo further differentiation into myofibroblasts after specific types of injuries. Myofibroblasts, which originate from keratocyte-derived or bone marrow-derived precursor cells, play a crucial role in the corneal stromal disorganized fibrotic extracellular matrix (ECM) [8]. When a lacerating injury occurs in the cornea, the contractile force generated by myofibroblasts aids in wound closure and maintains the mechanical integrity of the tissue [9].

Ideally, damaged stromal tissue needs to be repopulated by keratocytes that migrate from the surrounding stromal tissue. This migration should occur without generating strong contractile forces that could potentially disrupt the normal collagen architecture or lead to fibrotic ECM production and reduce the corneal transparency. While the process of corneal myofibroblast transformation of keratocytes has been extensively studied in animals [7,10,11,12,13], the process has not been definitively defined in humans.

In the current study, we investigated the pathophysiology of the human healing process in perforated corneal ulcers. We used transmission electron microscopy (TEM) and immunofluorescence (IF) to examine corneal tissue obtained from patients with healed perforated corneal ulcers (HPCUs) who underwent grafting procedures.

## 2. Materials and Methods

In this HPCU case series, all subjects (five cases) had a history of perforated corneal ulcers, which were treated until fully healed. Following ulcer recovery, all selected patients reported diminished vision due to corneal opacities ± leakage. To address this issue, keratoplasty was performed to eliminate the opacities and improve visual clarity. Operations were performed after 1–60 days of corneal perforations. During the surgery, excisions of the entire area of the healed corneal ulcer with surrounding corneal tissue and the attached iris were performed, with these excised tissues then cut into halves anteroposteriorly (from the epithelial side to the endothelial side) by the surgeon using a new sharp knife.

### 2.1. Transmission Electron Microscopy

Halved specimens were fixed with 4% glutaraldehyde in 0.05 M cacodylate buffer for 1 h, washed in 0.05 M cacodylate buffer (composed of sodium cacodylate 0.05 M as the primary buffer, distilled water as a solvent, and adjusted to pH 7.2–7.4 using HCl or NaOH) overnight, postfixed with osmium tetraoxide in veronal acetate buffer for 1 h, dehydrated in a series of ethanols and embedded in Luveac 812. Ultrathin sections were cut with a Porter-Blum MT2 microtome, stained with uranyl acetate and lead citrate, and examined with TEM (Hitachi H300, Hitachi, Ibaragi, Japan).

### 2.2. Immunofluorescence

The other halves of the surgical specimens were fixed in 10% formaldehyde and embedded in paraffin according to the standard protocol. Serial 4 µm histological sections were deparaffinized in xylene and hydrated in descending dilutions of ethanol. All samples were stained with hematoxylin and eosin (HE), and histological subtypes and the pathological stage were reconfirmed. For antigen retrieval, slides were placed in a water bath at 95 °C for 40 min in the ethylene diamine tetra acetic acid (Tris-EDTA) buffer (pH 9.0) or citrate buffer (pH 6.0) (Nichirei Bioscience, Tokyo, Japan). The slides were incubated at room temperature for 2 h and 1 h with the primary and the secondary antibodies, respectively, before they were mounted with phosphate-buffered saline (PBS) containing 10% glycerol and 1 µg/mL diamidino-2-phenylindole (DAPI). Images were taken using fluorescent microscopy (DM-6000B, Leica, Wetzlar, Germany) and captured with a digital camera (DFC-350 FX, Leica, Germany) attached to a fluorescent microscope.

The primary antibodies used were:

- Anti-CK14 antibody (1:100; BioLegend, San Diego, CA, USA)
- Anti-CK8/18 antibody (1:1000; Progen, Heidelberg, Germany)
- Anti-vimentin antibody (1:100; BioLegend, San Diego, CA, USA)
- Anti-(α-SMA) antibody (1:1000; Abcam Plc, Cambridge, UK)
- Anti-PCNA antibody (1:100; Abcam Plc, Cambridge, UK)
- Anti-phospho-histone H2AX antibody (1:200; Sigma-Aldrich, Burlington, VT, USA)
- Anti-Iba-1 antibody (1:500; Thermo Fisher Scientific, Waltham, MA, USA)
- Anti-CD45 antibody (1:50; Thermo Fisher Scientific, Waltham, MA, USA).

The secondary antibodies used were:

- Alexa Fluor 488-conjugated anti-mouse IgG antibody
- Alexa Fluor 555-conjugated anti-rabbit IgG antibody
- Alexa Fluor 647-conjugated anti-guinea pig antibody
- Alexa Fluor 647-conjugated anti-chicken antibody (all antibodies from Life Technologies, Carlsbad, CA, USA).

This study was approved by the Institutional Review Board of Nagasaki University (registration number: 24110703 on 7 November 2024) and was performed in line with the Declaration of Helsinki.

## 3. Results

The specimens ranged in size from 3 mm for most, up to one specimen at 8 mm.

### 3.1. Transmission Electron Microscopy

After the TEM examination, results showed that the corneal healing process could be categorized into three stages: early, intermediate, and late.

**Figure 1:**
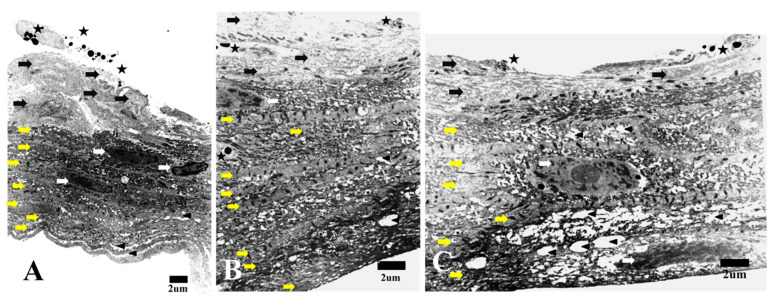
(**A**–**C**) TEM image showing the early stage of HPCUs in which many layered structures consist of two different types of cells. Cells include electron-lucent polygonal cells (black arrows) and elongated electron-dense cells (yellow arrows). Some elongated cells show mitotic changes (white arrows). Apoptotic changes can be frequently seen in superficial cells and infrequently in elongated cells (star = apoptotic body). Parallel layers of cells separated from each other with extracellular matrix (arrowhead) (bar = 2 µm).

**Figure 2:**
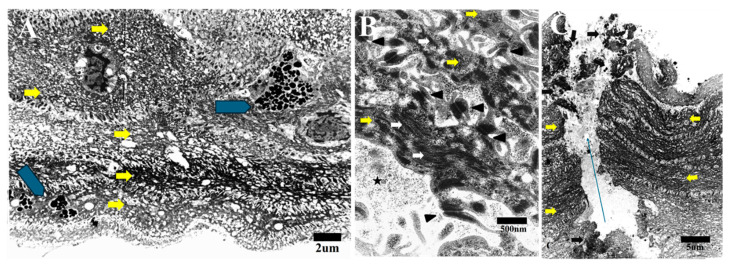
TEM image showing the early stage of HPCUs in which multiple macrophages (pentagon arrow), containing many apoptotic bodies and debris, are seen between elongated cells (yellow arrows) (bar = 2 µm) (**A**). Cells of the HPCU (yellow arrows) are connected to each other with multiple gap junctions (arrowheads) and contain haphazard distribution of stress fibers (white arrows) (star = ECM) (bar = 500 nm) (**B**). The parallel layers of cells (yellow arrows) sometimes show anteroposterior space (micro perforation = long arrow) with adjacent degenerated cells (black arrows) (bar = 5 µm) (**C**).

During the early stage, TEM examination of HPCUs displayed numerous layered structures with two distinct cell types attached. Cells include superficial polygonal electron lucent cells and elongated relatively electron dense cells (Figure 1). Polygonal cells frequently exhibited disintegrated cell membranes and dispersed cellular debris, with apoptotic changes like condensed chromatin bodies (apoptotic bodies) (Figure 1). In addition, there were a few elongated cells that displayed chromatin condensation and cytoplasmic shrinkage, which indicated apoptotic changes (Figure 1B). In contrast, mitotic changes were frequently seen in the elongated cells, as shown in Figure 1. Furthermore, we also detected some macrophages that contained many apoptotic bodies and debris between the elongated cells during this early stage (Figure 2A).

All the cells were interconnected through numerous gaps or adherens junctions, with intercalation of the cell membranes among the neighboring cells (Figure 2B). The cells were organized in parallel layers, especially the elongated cells with deposits of ground substance in the ECM. All the cells during this stage did not exhibit any distinct separation between the two types of cells.

Both cell types contained intracytoplasmic microfilaments that were haphazardly distributed, especially in the elongated cells (Figure 2B). There was no presence of endothelial cells or Descemet’s membrane (DM) observed throughout the specimen. Remarkably, corneal full thickness micro-perforations were observed and which featured minute anteroposterior spaces between the cells. Degenerative cell remnants were also found along the sides of these tiny spaces (Figure 2C).

**Figure 3:**
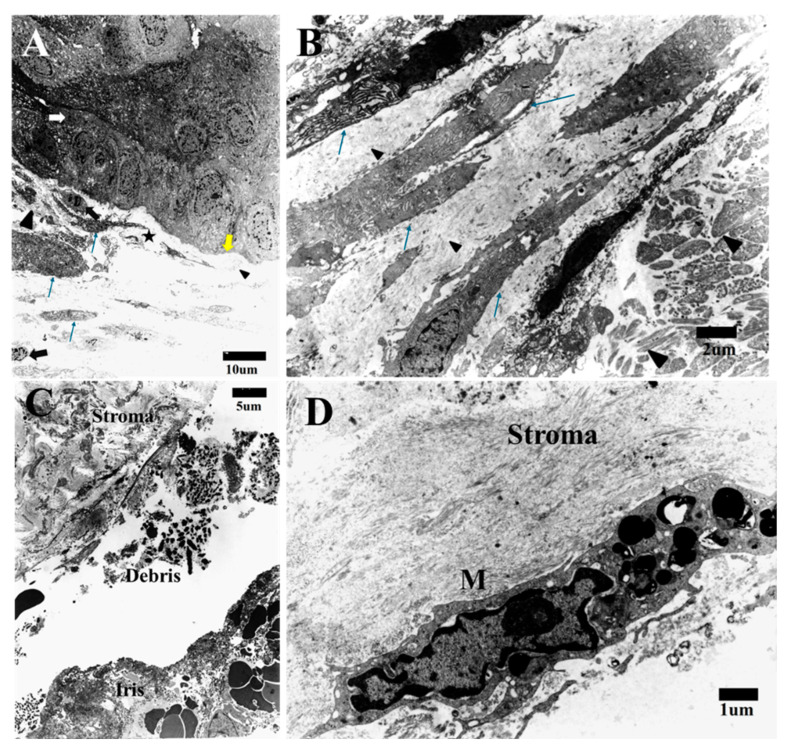
TEM image showing intermediate stage of HPCUs in which the epithelium (white arrow) matures and separates from the stroma with a basement membrane (yellow arrow). Elongated active cells (thin long arrows) are seen in the stroma and some cells show shrinkage and condensation of their chromatin (apoptotic changes) (black arrows) (star = early BM; small arrowhead = fine fibers, and big arrowhead = bundles of fibers) (bar = 10 μm) (**A**). TEM image showing stroma with multiple elongated cells (thin long arrows) containing rough endoplasmic reticulum and other organelles. ECM contains either fine fibrils (small arrowhead) or densely packed collagen bundles (big arrowhead) (bar = 2 μm) (**B**). TEM image showing that the stroma is adjacent to iris tissue with debris intervening with no endothelial cells or DM (bar = 5 μm) (**C**). Large macrophage (M) containing apoptotic bodies and debris can be seen in the stroma (bar = 1 μm) (**D**).

In the intermediate stage, the cornea consisted of three layers [epithelium, Bowman’s layer (BL), and stroma] (Figure 3). The epithelium consisted of five to six layers of normal corneal epithelium with a distinctive basement membrane. During this stage, the BL contains some elongated cells with adjacent irregular and non-condensed collagen fibers (Figure 3A). The stromal elongated cells were randomly arranged and deposited microfilaments throughout the stroma in an irregular manner, leaving spaces between the cells. These elongated cells featured prominent rough endoplasmic reticulum, indicating high activation (Figure 3B). The deposited microfilaments appeared either as fine fibrils (primitive collagen fibers) or densely packed collagen bundles. In both cases, their arrangements were disorganized (Figure 3B).

There was neither DM nor an endothelial layer, and the stroma was adjacent to the iris tissue with debris intervening (Figure 3C). Macrophages with intracellular dense chromatin bodies and debris were scattered in the stroma (Figure 3D).

**Figure 4:**
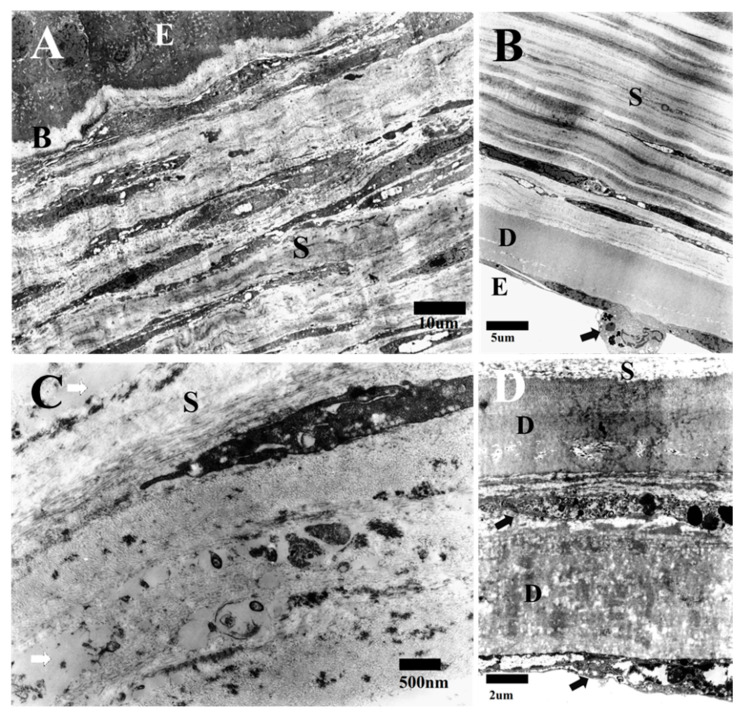
TEM image showing the outer half of the stroma during the late stage where stromal cells are elongated and arranged in a uniform pattern parallel to the corneal surface. (E = epithelium; B = Bowman’s layer, and S = stroma) (bar = 10 μm) (**A**). The inner part of the stroma (S) shows that collagen fibers are organized into lamellae, with each layer oriented perpendicular to the adjacent one, with flattened cells interspersed between them (D = DM; E = endothelium; and black arrow = macrophage) (bar = 5 μm) (**B**). Stroma (S) showing that collagen fibers are organized into lamellae with interstitial spaces occasionally containing ground substances (white arrows) (bar = 500 nm) (**C**). TEM image shows duplication of DM (D) with duplication of endothelial cells (black arrows) (S = stroma) (bar = 2 μm) (**D**).

During the late stage, the cornea displayed its typical five layers following the migration of the endothelial cells over the stroma and the development of the DM. BL appeared normal and was devoid of any cells, similar to that observed in the intermediate stage (Figure 4A). The stromal disordered cells became elongated, arranging themselves in a uniform pattern parallel to the corneal surface (Figure 4A,B). The elongated cells featured a prominent rough endoplasmic reticulum, especially in the outer half of the stroma, and contained moderately electron-dense organelles with intracytoplasmic vacuoles, which indicated high activation (Figure 4A). The stroma thickened due to the creation of more orderly lamellae of fibers, with flattened cells interspersed between them. The collagen fibers were organized into lamellae, with each layer oriented perpendicular to the adjacent one (Figure 4B,C). Although the fibers appeared to be more systematically organized, we observed that the interstitial spaces occasionally contained ground substances (Figure 4C).

The endothelial cells appeared to cover the inner side of the HPCU with the deposition of DM (Figure 4B). In addition, macrophages were seen among endothelial cells that contained apoptotic bodies and debris (Figure 4B). During this late stage, we sometimes found duplication of the DM with duplication of the endothelial cells (Figure 4D).

### 3.2. Immunofluorescence

**Figure 5:**
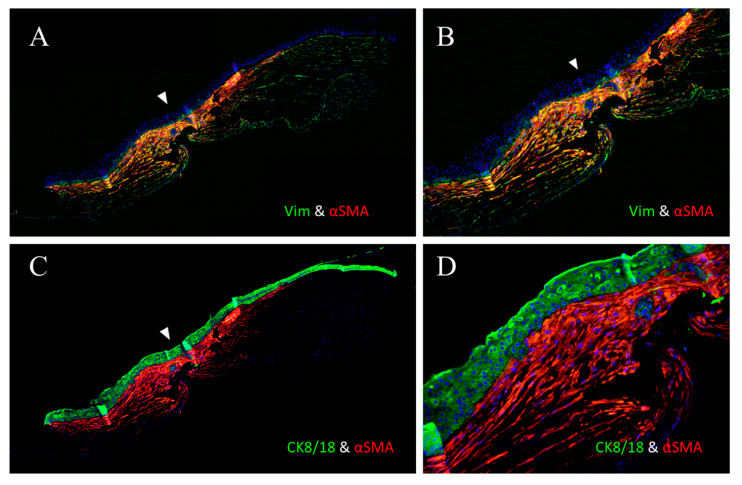
IHF image showing the corneal tissue at the HPCU during low (**A**,**C**) and high (**B**,**D**) magnification. This shows that the cornea is very thin at the HPCU (arrowhead), with two types of cells, including the superficial polygonal cells that are continuous with the corneal epithelium, and the deeper cells that are continuous with the corneal stroma. The stromal cells showed both vimentin^+^ (green) and α-SMA^+^ (red) myofibroblasts and these cells were very condensed at and near the HPCU, while they decreased when moving from the HPCU (**A**,**B**). Double IHF staining with CK8/18 and α-SMA showed that the superficial epithelial cells were CK8/18^+^ (green), while deep stromal cells were α-SMA^+^ (red) (**C**,**D**). ((**A**,**C**) magnification = ×40, (**B**,**D**) magnification = ×200).

At the site of the HPCU, we detected that the cornea was very thin with two types of cells. Superficial polygonal cells were continuous with the corneal epithelium, while the deeper cells were continuous with the corneal stroma. The stromal cells exhibited both vimentin^+^ and α-SMA^+^ myofibroblasts (Figure 5A,B). These cells were highly condensed at and near the HPCU, becoming decreased in areas away from the HPCU (Figure 5A,B).

Double IF staining with CK8/18 and α-SMA showed that the superficial epithelial cells were CK8/18^+^, while the deep stromal cells were α-SMA^+^ (Figure 5C,D).

**Figure 6:**
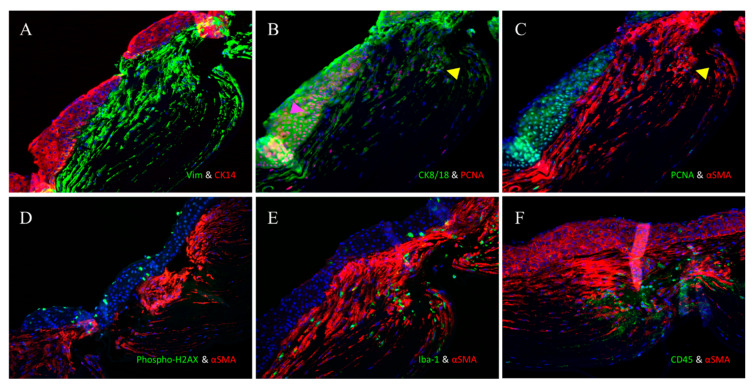
The superficial epithelial cells exhibited staining with anti-CK14 (red), while the stromal cells exhibited staining with anti-vimentin (green) (**A**). There was staining of the superficial epithelial cells with anti-CK8/18 (green), while some cells of the epithelium exhibited staining with anti-PCNA (pink arrowhead) along with the stromal cells (red) (yellow arrowhead) (**B**). Double staining with anti-PCNA and anti-α-SMA confirmed that some of the myofibroblasts had undergone mitotic changes (yellow arrowhead) (**C**). Double staining with anti-phospho-histone H2AX and anti-α-SMA showed that while apoptosis was frequently seen in the epithelium, it was rarely found in the myofibroblasts (**D**). Double staining using anti-Iba-1 and anti-α-SMA showed the presence of numerous macrophages in the α-SMA^+^-rich region, with a few positive cells in the epithelial region (**E**). Many cells in the α-SMA^+^-rich region were also CD45^+^ (**F**). (Magnification = ×200).

Double IF staining with CK14 and vimentin showed that the superficial epithelial cells were CK14^+^, while the deep stromal cells were vimentin^+^ (Figure 6A).

The PCNA antibody, which targets proliferating cell nuclear antigen, is a highly sensitive marker for cell proliferation and mitosis. Mitotic cells are typically observed in the epithelium as part of the normal life cycle of epithelial cells in unwounded corneas. When double staining with CK8/18 and the PCNA antibody was performed, multiple stromal and many epithelial cells were found to be undergoing mitosis (PCNA^+^) (Figure 6B). Double staining with the PCNA antibody and α-SMA confirmed that some myofibroblasts had undergone mitotic changes (Figure 6C).

The anti-phosphorylated histone H2AX antibody targets the phosphorylated form of histone H2AX at serine 139 (often referred to as γ-H2AX). This phosphorylation event is widely used in apoptosis studies due to its high sensitivity in detecting DNA double-strand breaks caused by apoptosis. Double staining with an anti-phospho-histone H2AX antibody and α-SMA showed that apoptosis was frequently seen in the epithelium and rarely found in myofibroblasts (Figure 6D).

Additionally, we also conducted double staining using the anti-Iba-1 antibody, which is specific for macrophages, and α-SMA. This confirmed the presence of numerous macrophages in the α-SMA^+^ cell-rich region, with only a few positive cells observed in the epithelial region (Figure 6E).

Finally, when the double IF for α-SMA and CD45 were performed, it was found that many of the α-SMA^+^-rich regions were CD45^+^ (Figure 6F).

## 4. Discussion

Corneal injury can potentially lead to ulceration, which remains a major health concern in ocular surface diseases. While in vitro studies often use animal corneas, these models may not fully represent the human corneal repair process due to the structural and physiological differences between human and animal eyes [14]. To the best of our knowledge, this study is among the few that have sought to assess the healing process of perforated corneal ulcers in humans.

The corneal epithelium, which acts as the outer protective layer, continuously renews itself with an impressive regenerative ability. Stem cells located in the limbal palisades migrate towards the corneal center, where they differentiate into transient amplifying cells and basal cells. This renewal process involves not only the vertical movement of the differentiating cells from the deeper to the more superficial layers, but also involves the centripetal migration of stem cells from the limbus to the central cornea during differentiation [15].

After epithelial damage, the corneal stroma undergoes keratocyte apoptosis beneath the wound, which is triggered by cytokines that are released from the damaged epithelial cells. Consequently, the area beneath the wound becomes nearly devoid of cells (acellular zone) [16,17]. Meanwhile, keratocytes at the wound’s periphery change their phenotype, becoming activated and transforming into motile corneal fibroblasts [18]. In a rabbit model of transcorneal freeze injury, fibroblasts were found to organize into connected streams that were aligned parallel to the collagen lamellae. This alignment suggests that the lamellae play a role in providing “contact guidance” for fibroblast migration [19].

In earlier studies, researchers observed there was a similar migration pattern of corneal fibroblasts that invaded 3D fibrin matrices using a nested matrix model. In this model, the fibroblasts extend their leading edge into the fibrin while maintaining connections with the cells behind them. As they follow similar paths, they form elongated streams of interconnected cells [20]. Lateral protrusions between adjacent cells interconnect, forming a mesh-like structure. In the nested matrix model, fibrin fibrils are randomly organized [19].

Upon reaching the wound bed, fibroblasts begin expressing α-SMA and desmin, upregulate vimentin expression, and transform into highly motile and contractile myofibroblasts. These myofibroblasts are essential for remodeling the wound ECM and contracting the wound [21]. They also deposit provisional ECM rich in fibronectin and some other proteins, including tenascin-C and type III collagen [22]. Myofibroblasts produce contractile forces to close the wound gap, and the expression of α-SMA is directly correlated with the corneal wound contraction [23,24,25].

In our current study, we detected two types of cells in the early stage of healing in the area of HPCUs. Superficial polygonal electron lucent cells were in contact with many parallel layers of elongated electron-dense cells. All these cells were connected to each other, both with many gap or adherens junctions, and there was intercalation of the cell membranes with adjacent cells. The elongated cells show a haphazard distribution of microfilaments, and each layer was separated from the adjacent layers with ECM. The morphology of the superficial polygonal electron lucent cells was consistent with epithelial cells, while the elongated cells were consistent with that of migratory corneal fibroblasts and interestingly, these cells were generally arranged in long, parallel lines. The IF labeling confirmed that these were fibroblastic cells, as indicated by α-SMA (intracellular stress fibers) and vimentin expression.

We further determined that the superficial polygonal cells were epithelial in nature and observed apoptotic changes in some of these cells through TEM and IF analysis. However, we did not detect an acellular zone formed by the apoptosis of quiescent keratocytes following corneal injury. Instead, we confirmed the activation of keratocytes and their transformation into activated fibroblasts, which then organized themselves into parallel layers beneath the epithelial layer. We were unable to confirm the presence of any acellular zone or determine if we perhaps missed it due to its occurrence at a very early stage.

Interestingly, we observed full-thickness anteroposterior micro-perforation in the cornea, which was characterized by tiny anteroposterior space between the cells. We believe this space was not an artifact, as it was encircled by apoptotic cells and debris. Its presence may provide insight into why patients continued to experience tearing despite the complete healing of their perforated corneal ulcer.

Myofibroblasts are fibroblastic cells that can be generated from either keratocyte-derived or bone marrow-derived precursor cells [11,26,27,28]. Epithelial–stromal interactions modulate the generation of corneal myofibroblasts and the development of stromal opacity [29,30]. The epithelial basement membrane plays a critical role in modulating myofibroblast development and persistence [31,32].

Myofibroblasts exhibit variable cell phenotypes based on immunohistochemical staining of intermediate filament proteins [21,33]. The cytoskeleton system in the human cornea is composed of intermediate filament proteins, such as vimentin and desmin, as well as actin microfilaments, microtubules, and their associated proteins. These components interact to create a meshwork essential for the normal growth, differentiation, integrity, and function of corneal cells [34]. Vimentin is a major class of intermediate filament proteins in mesenchymal cells such as fibroblasts, vascular endothelial cells, and adipocytes [35].

Subsequently, fibroblasts proliferate to repopulate the wound site. During stromal healing, their migration and activation are assumed to be mediated by several growth factors, including transforming growth factor beta (TGFβ) and platelet-derived growth factor (PDGF) [19,36,37].

Throughout the entire repair process, inflammatory cells, especially macrophages, play a crucial role. These macrophages secrete TGF-β1 and proteases, which help coordinate the interactions between myofibroblasts and the ECM. Specifically, macrophages in close proximity to contractile myofibroblasts influence both the composition and the biophysical properties of the ECM [38,39]. Similarly to the findings reported from other studies, our current results also showed that macrophages were closely associated with myofibroblasts.

In our current study, the epithelium formed its basement membrane and became separated from the stroma during the intermediate stage of healing. The stromal elongated cells were arranged randomly and deposited microfilaments throughout the stroma in an irregular manner, leaving spaces between the cells. These elongated cells featured prominent rough endoplasmic reticulum, which indicates high activation. The deposited microfilaments appeared either as fine fibrils (primitive collagen fibers) or densely packed collagen bundles. In both cases, their arrangement was disorganized. We concur with previous studies that the disorganized ECM and the irregular distribution of new fibrils are the primary causes of corneal haze or opacity that are observed during the healing process of corneal perforation. We did not detect any endothelial cells or DM at the HPCU during the early or intermediate stages of corneal regeneration.

During endothelial wound healing, migrating cells temporarily acquire a fibroblastic morphology and develop actin stress fibers, a process that is consistent with endothelial mesenchymal transformation (EnMT) [40,41]. EnMT to myofibroblasts occurs at the migrating front, where cells lose the tight junction protein ZO-1 and start expressing α-SMA [42]. The consequences of EnMT are considered undesirable, as this can lead to the loss of endothelial cells and corneal opacification due to ECM deposition. Additionally, EnMT is observed in ex vivo-cultured endothelial cells, and has been shown to result in the loss of endothelial cell characteristics during attempts to bioengineer endothelial grafts [43].

In our current study, IF showed that most of the elongated flat cells in the TEM pictures were α-SMA^+^ and vimentin^+^ cells, denoting that these were myofibroblasts. The origin of these myofibroblasts can be explained either from stromal keratocytes, EnMT, or bone marrow-derived cells in a separate or combined condition. We propose that some of the myofibroblasts originate from stromal keratocytes, while others arise from the bone marrow-derived precursor cells (CD45^+^ cells with IF), rather than from the EnMT process. In addition, among all of the corneal layers, the human corneal endothelium has the lowest mitotic activity and the lowest regenerative capacity in contrast to the animal endothelial cells. We also assumed that due to the activity of the myofibroblasts, corneal stromal fibrosis was associated with the occurrence of the opacity, and thus, the lamellar keratoplasty was a necessity in our current cases.

Although the stromal cell populations are rapidly replenished, the main challenge in stromal wound healing is to restore the highly regular collagen organization that is initially lost at the injury site. This remodeling process and the restoration of normal transparency can take several years to accomplish [44]. Among all of the corneal layers, the endothelium exhibits the lowest mitotic activity and regenerative capacity. In order to close the small endothelial ruptures, the remaining endothelial cells need to migrate and enlarge to remodel the monolayer. As a result, these can then re-establish the barrier function and resume their pumping activity [45].

In the final phase of our current study, we found that the endothelial cells from either side of the wound moved to cover the inner side of the defect, thereby secreting a new DM. This process could explain the presence of the DM duplication that was observed in certain sections of the HPCU, and which likely resulted from the overlapping migration of endothelial cells from both wound edges, leading to the duplication of DM. Additionally, there was significant thickening of the stroma due to the deposition of collagen fibers arranged in parallel lamellae. Keratocytes resumed their typical elongated shape between the corneal lamellae. Throughout this process, the disorganized repair matrix was gradually replaced by the regular corneal ECM.

This study has the potential to aid clinicians and researchers in making timely interventions based on healing stages, selecting the most suitable medical or surgical treatments, improving patient counseling and adherence, and driving further research into healing enhancers.

## 5. Conclusions

Epithelial cells and fibroblasts participate in the initial and intermediate stages, and form the outer three layers of the cornea (including the main bulk of the stroma). During the final stage, the endothelial cells migrate from the adjacent area of the perforation to cover the inner side of the wound, thereby forming a new DM. The healing of corneal wounds is a complex process that includes apoptosis, migration, mitosis, and differentiation of different cells with the remodeling of the extracellular matrix.

## Data Availability

Data is provided within the manuscript.
